# Effects of soil C:N:P stoichiometry on biomass allocation in the alpine and arid steppe systems

**DOI:** 10.1002/ece3.2710

**Published:** 2017-02-01

**Authors:** Xiaodan Wang, Xingxing Ma, Yan Yan

**Affiliations:** ^1^ Institute of Mountain Hazards and EnvironmentChinese Academy of SciencesChengduChina

**Keywords:** biomass allocation, soil stoichiometry, soil–plant interaction, Tibetan Plateau

## Abstract

Soil nutrients strongly influence biomass allocation. However, few studies have examined patterns induced by soil C:N:P stoichiometry in alpine and arid ecosystems. Samples were collected from 44 sites with similar elevation along the 220‐km transect at spatial intervals of 5 km along the northern Tibetan Plateau. Aboveground biomass (AGB) levels were measured by cutting a sward in each plot. Belowground biomass (BGB) levels were collected from soil pits in a block of 1 m × 1 m in actual root depth. We observed significant decreases in AGB and BGB levels but increases in the BGB:AGB ratio with increases in latitude. Although soil is characterized by structural complexity and spatial heterogeneity, we observed remarkably consistent C:N:P ratios within the cryic aridisols. We observed significant nonlinear relationships between the soil N:P and BGB:AGB ratios. The critical N:P ratio in soils was measured at approximately 2.0, above which the probability of BGB:AGB response to nutrient availability is small. These findings serve as interesting contributions to the global data pool on arid plant stoichiometry, given the previously limited knowledge regarding high‐altitude regions.

## 1 Introduction

Biomass allocation is an important variable in the terrestrial ecosystem carbon cycle. Plants may change their allocation patterns in response to the environment (Reich et al., 2003). Quantitative understandings of the biomass allocation patterns are of fundamental importance to ecological management (Niklas, 1994). Several studies have identified the functional equilibrium hypothesis of biomass allocation patterns in various plants (Cannell & Dewar, 1994; Reich et al., 2003; Wilson, 1988). There are basically two schools of thought (functional equilibrium and allometric relationship) on the description and analysis of plant organ allocation. The functional equilibrium hypothesis suggests that plants respond to variations in environmental conditions by allocating biomass at any given time across various organs to capture nutrients, water, and light and thus to maximize growth rates (Bloom, Chapin, & Mooney, 1985; Evans, 1972; Marcelis, Heuvelink, & Goudriaan, 1998). Based on this hypothesis, relative plant growth rates are then determined by the product of the net nitrogen (N) uptake rate per unit of root mass, by plant N concentrations and by the fraction of biomass invested in roots (Garnier, 1991; McConnaughay & Coleman, 1999). The second perspective is that of the allometric approach. Rather than considering ratios at specific times, it describes the overall relationship between the total amount of one organ (for example, the shoot mass) and another (for example, the root mass; Niklas, 1994).

Over the last 20 years, experimental evidence on relationships between soil, water, and organismic C:N:P ratios and on essential ecological traits has expanded, and these relationships now play a central role in ecological research (Raubenheimer & Simpson, 2004; Sterner & Hessen, 1994). Several authors have reported that plants and soil microorganisms maintain a certain stoichiometric balance of C:N:P elements to function properly (Cleveland & Liptzin, 2007; Redfield, 1958; Reiners, 1986). Changing the N:P ratio by, for instance, adding one element may induce different responses in different species, which may then affect element availability levels while altering patterns of biomass allocation (Fujita, de Ruiter, Wassen, & Heil, 2010; Gusewell, 2004). However, a few studies have found extensive variations in C:N, C:P, and N:P ratios. For example, in terrestrial plants, C:N values were found to range from c. 5 to >100, and C:P values were found to range from <250 to >3500 (Elser, 2002; Elser et al., 2000). Other authors have found that variations in N:P are not related to the total amount of standing biomass, denoting that vegetation types ranging from grassland, shrubland, and forest do not differ systematically with respect to N:P ratios (Kerkhoff, Enquist, Elser, & Fagan, 2005). Strategies of biomass allocation remain contested in the field of plant ecology (Xiao et al., 2003; Yang, Fang, Ji, & Han, 2009).

Alpine plants have adapted to low temperatures and are thus expected to have developed unique survival mechanisms, enhancing the value of regional and global studies that consider such plants (Chapin & Körner, 1995; Wang et al., 2011). High‐altitude areas tend to be indirectly N deficient due to the presence of low temperatures and soil erosion induced by freeze–thaw circulation (Chapin, 1983; Grogan, Michelsen, Ambus, & Jonasson, 2004; van Heerwaarden, Toet, & Aerts, 2003), whereas tropical and subtropical vegetation areas tend to exhibit low P but high N availability levels (Gusewell, 2004; McGroddy, Daufresne, & Hedin, 2004). However, some authors have found life forms and genus identities to constitute main factors that affect foliar N levels and C:N ratios, but that low‐temperature limitations on organic matter mineralization and N availability levels in soils cannot explain low leaf N levels in cold environments at the biome scale (He et al., 2006). This dispute is compounded by challenges of ensuring standard data collection practices. The Tibetan Plateau, with an average elevation exceeding 4,000 m, represents one of the largest alpine and arid steppes in the world (Wang, Yan, & Cao, 2012). With the exception of general floristic and soil accounts in some areas, virtually no information exists on any aspect of the structure and functioning of the Tibetan alpine and arid steppe on a global scale (Ram, Singh, & Singh, 1989; Reich & Oleksyn, 2004; Wright et al., 2004). In addition, several previous studies have focused on leaf stoichiometry features rather than on entire individuals. This focus appears insufficient to understanding biomass allocation mechanisms, as most growth rate hypotheses focus on whole organisms. C:N:P measurements conducted at the whole‐plant level will allow us to compare differences in plant and animal stoichiometry levels (Chapin & Körner, 1995; Elser, 2002; Elser et al., 2000).

In this study, we evaluate biomass, carbon, nitrogen, and phosphorus features of a high‐altitude steppe along the northern Tibetan Plateau. Our objectives are (1) to explore interactions between carbon, nitrogen, and phosphorus at the community level in soils and plants; (2) to detect patterns of biomass allocation in the alpine steppe along the designated latitude; and (3) to clarify the relationship between above‐ and belowground biomasses and soil C:N:P stoichiometry.

## 2 Materials and Methods

### 2.1 Study area

**Figure 1:**
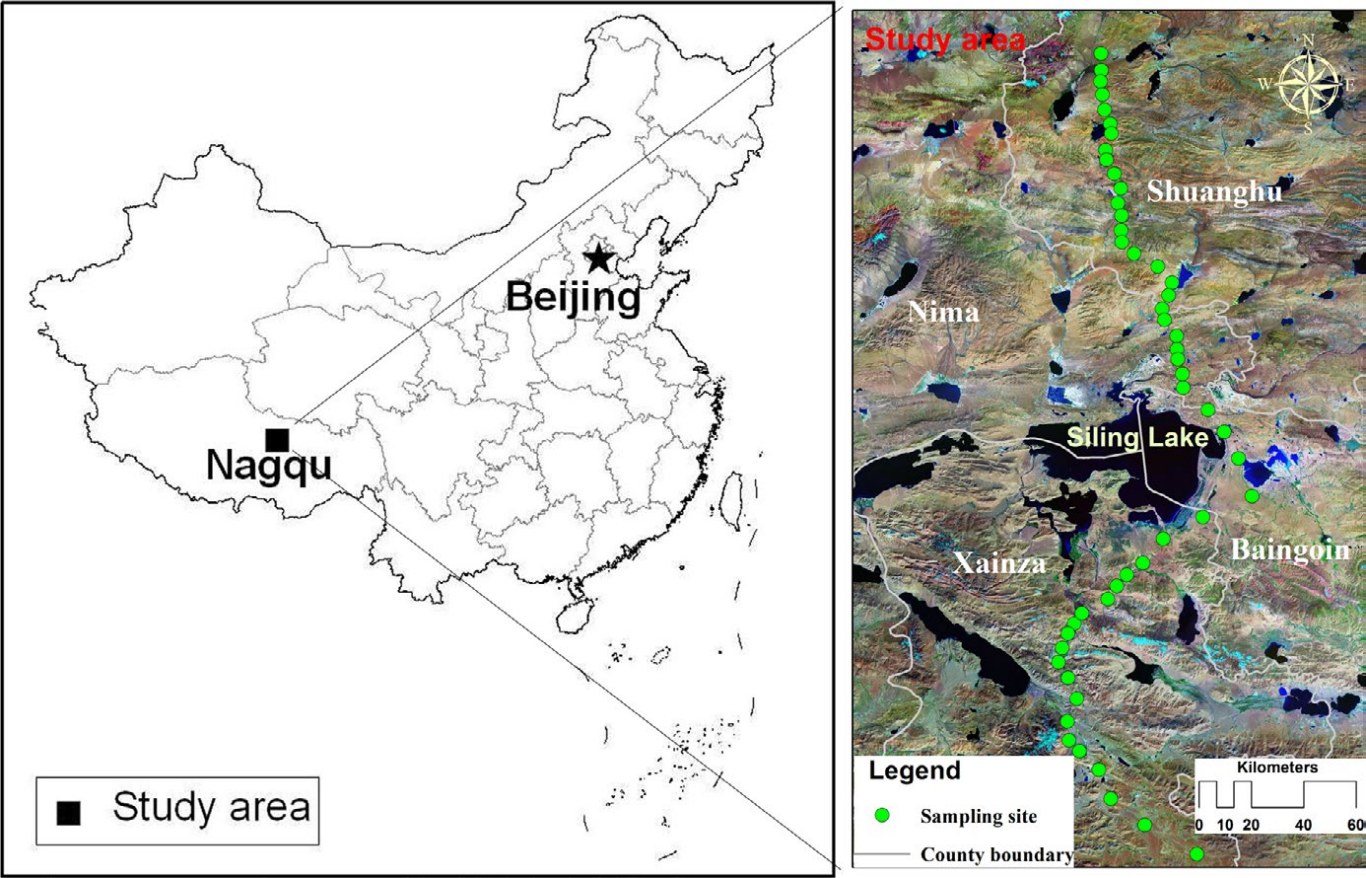
Location of the study area. The background color map is Landsat Thematic Mapper (TM) satellite imagery, and the circles denote the sampling sites

The study was conducted on an alpine and arid steppe located along the 300‐km transect in Nagqu prefecture (83°52′–95°01′E, 29°56′–36°41′N, average altitude of ~4,600 m) of the northern Tibetan Plateau, China (Figure 1). The mean annual temperature of this transect is approximately −0.8°C, with monthly averages ranging from a minimum of −11.3°C in January to a maximum of 8.3°C in July. Annual rainfall levels are <300 mm, with degrees of high variation. Winds can exceed 17 m/s on as many as 150 days/year, and maximum wind velocities can reach as high as 40 m/s. The climate becomes drier and colder in the northern region along the transect. Soils in the area are classified as cryic aridisols under the FAO–UNESCO system and are characterized by youth, coarse textures, and loose structures. The soil parent materials were from the heavily weathered limestone. Local steppe vegetation is mainly dominated by *Stipa glareosa P. Smirn* and *Carex moorcroftii Falc. Ex Boott*, which are associated with *Orinus thoroldii S. Bor* and *Leontopodium nanum* (Wang et al., 2012).

### 2.2 Sample collection

Samples were collected from 44 sites along the transect at spatial intervals of 5 km in July of 2013 (Figure 1). Three plots (replicates) of 1 m × 1 m were randomly assigned to each sampling site (area 3 m^2^). Aboveground fresh biomass levels were measured by cutting a sward (1 m × 1 m) in each plot. All standing live and dead culms were removed and placed in plastic bags. The BGB was collected from three soil pits in a block of 1 m × 1 m of actual root depth. Soil cores (depth 50 cm and diameter 5 cm) were taken in the middle of each plot after the aboveground biomass sample was collected. All 44 samples were transported to the laboratory. Root samples were cleaned in deionized water and residual soils were removed by using a 0.5‐mm sieve. The plant samples were then oven‐dried at 70°C for 72 hr and weighed to the nearest 0.01 g. The soil samples were air‐dried, hand‐sieved through a 2‐mm screen, and handpicked for the extraction of fine roots for physical and chemical analysis.

### 2.3 Analytical methods

The soil organic carbon (C) analysis was conducted using the traditional wet digestion method, total nitrogen (N) and total phosphorus (P) were extracted from the soil using the semi‐micro Kjeldahl method, and then, the concentration was detected on a spectrophotometer (Peri & Lasagno, 2010). We measured the whole‐plant concentrations of C, N, and P. Plant carbon was extracted by the traditional dry combustion method, N was extracted using the Kjeldahl method, and then, the concentration was detected on a spectrophotometer. Plant tissue P was extracted from the tissue using a digestion method, and P levels were determined via atomic emission spectrometry (ICP‐AES). All ratios were calculated on a mass basis.

### 2.4 Statistics

SPSS 14.0 for Windows (SPSS Inc., Chicago, IL, USA) was used for the statistical analyses. A linear regression analysis with soil nutrient elements as the independent parameter was conducted to test whether the soil C:N:P stoichiometry affected biomass allocation (BGB:AGB) levels in the alpine and arid steppe. Prior to all of the analyses, nutrient ratios were log_10_ transformed to improve the distribution and homogeneity of variance, but all means and standard errors were back transformed into original units (Cleveland & Liptzin, 2007).

## 3 Results

### 3.1 Biomass allocation of alpine and arid steppe

**Figure 2:**
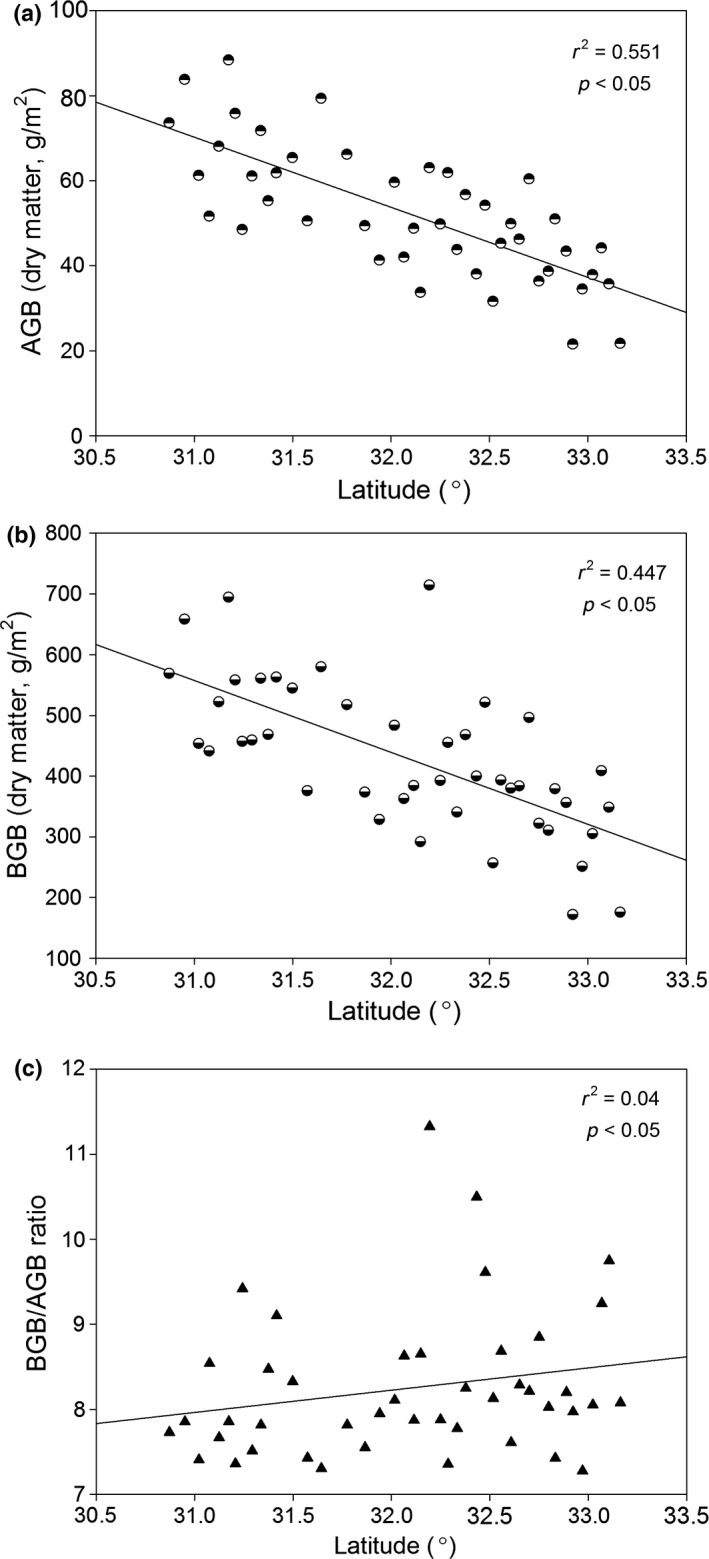
Biomass changes with latitude (AGB, aboveground biomass; BGB, belowground biomass) (*p* < 0.05)

Total biomass levels ranged from 193.7 to 783.2 g/m^2^ with a mean of 495.8 g/m^2^ (Table 1). The aboveground biomass (AGB) level, including stems and leaves, ranged from 21.6 to 102.6 g/m^2^ with a mean of 56.8 g/m^2^, and it constituted 11.5% of the total biomass (Table 1). The belowground biomass (BGB) ranged from 172.1 to 714.7 g/m^2^ with a mean of 439.0 g/m^2^ and represented 88.5% of the total biomass (Table 1). A significant negative correlation was found between the latitude and AGB, BGB (*p* < .05, *r* ^2^ = 0.551, and 0.447, respectively, Figure 2a,b). The BGB:AGB ratio in the alpine and arid steppe varied from 7.3 to 11.3 with a mean of 8.2. Figure 2c shows that the BGB:AGB ratio was positively affected by the latitude (*p* < .05, *r* ^2^ = .04).

**Table 1:** Above‐ and belowground biomasses of *Stipa glareosa P. Smirn* and *Carex moorcroftii Falc. Ex Boott* community in an alpine steppe along a latitude transect

	Descriptive statistics			
	Minimum	Maximum	Mean	Percent (%)^a^
AGB (g/m^2^)	21.6	88.4	52.4 ± 15.5	10.9
BGB (g/m^2^)	172.1	714.7	429.3 ± 123.5	89.1
Total biomass (g/m^2^)	193.7	783.2	481.7 ± 138.0	100
BGB/AGB ratio	7.3	11.3	8.2 ± 0.9	—

AGB, aboveground biomass (dry matter weight); BGB, belowground biomass (dry matter weight).

a In this column, percentage values are the ratios of AGB and BGB to the total biomass, respectively.

### 3.2 Carbon, nitrogen, and phosphorus in soils and plants

**Figure 3:**
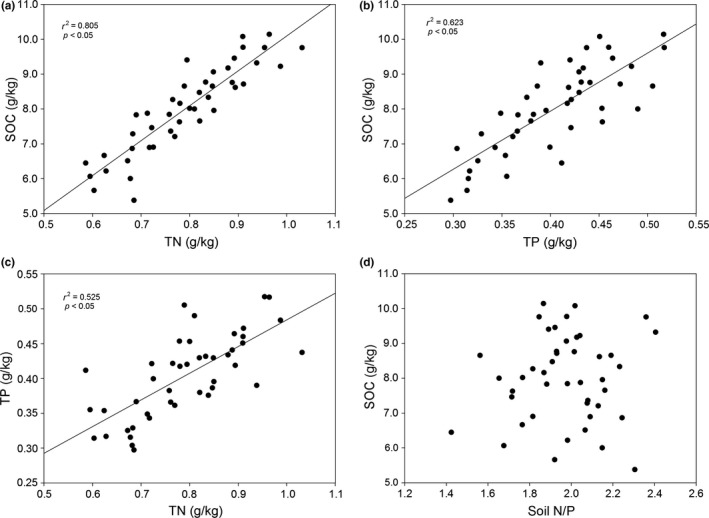
Relationships between C, N, and P in the soil (*p* < 0.05)

As shown in Table 2, average C, N, and P levels in the soil were recorded 8.0, 0.8, and 0.4 g/kg, respectively. Soil C levels varied from 5.4 to 10.1 g/kg, and total soil N levels varied from 0.6 to 1.0 g/kg. However, although soil C, N, and P content levels were variable, our analysis shows that the total soil C:N, C:P ratios were remarkably constrained (Figure 3, Table 2). The soil C:N ratios varied from 7.9 to 11.8, and the N:P ratios varied from 1.4 to 2.4.

**Table 2:** C, N, and P elements of *Stipa glareosa P. Smirn* and *Carex moorcroftii Falc. Ex Boott* community in an alpine steppe along a latitude transect

	Soil (0–30 cm)			Plant (root, stem, leaf)		
	Minimum	Maximum	Mean	Minimum	Maximum	Mean
OC (g/kg)	5.4	10.1	8.0 ± 1.3	403.9	526.0	466.8 ± 29.3
TN (g/kg)	0.6	1.0	0.8 ± 0.1	7.2	14.1	10.8 ± 2.1
TP (g/kg)	0.3	0.5	0.4 ± 0.1	2.1	4.2	3.2 ± 0.6
C/N	7.9	11.8	10.1 ± 0.7	31.9	67.1	44.9 ± 9.6
C/P	15.7	23.9	19.9 ± 1.9	114.7	224.8	150.3 ± 24.9
N/P	1.4	2.4	2.0 ± 0.2	1.9	5.2	3.5 ± 0.9

OC, organic carbon; TN, total nitrogen; TP, total phosphorus.

**Figure 4:**
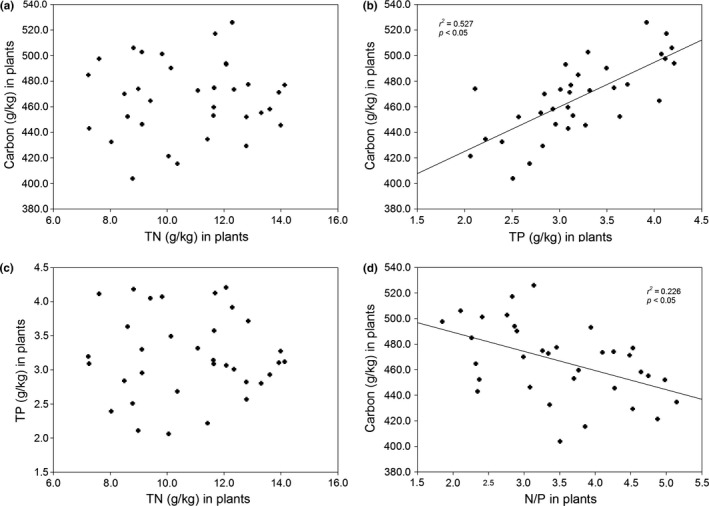
Relationships between C, N, and P in the plants (*p* < 0.05)

Mean C, N, and P contents in the *Stipa* community were recorded at 466.8, 10.8, and 3.2 g/kg, respectively (Table 2). At the community level, biomass C:N, C:P, and N:P mass ratios were recorded at 44.9, 150.3, and 3.5, respectively (Table 2). P and C levels typically correlated positively with one another (*r* ^2^ = .527, *p* < .05, Figure 4b). N:P and C tended to correlate negatively (*r* ^2^ = .226, *p* < .05, Figure 4d). However, no significant relationship was found between C and N (Figure 4a) or between N and P (Figure 4c).

### 3.3 Effects of soil N:P ratios on biomass allocation

**Figure 5:**
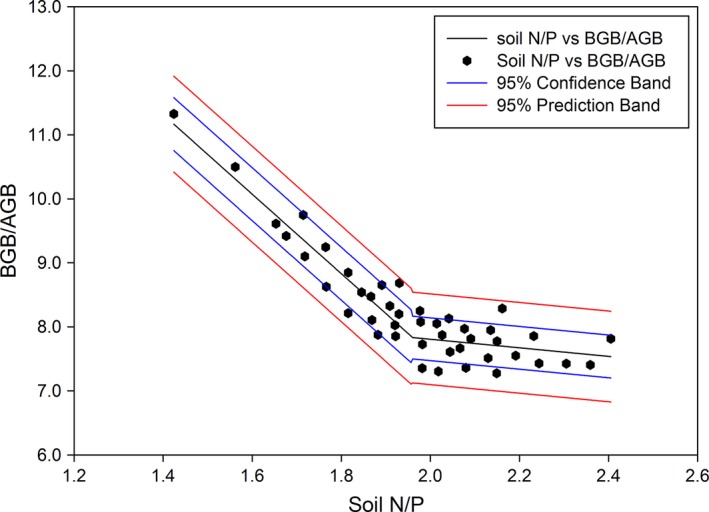
The fitted curve shows the relationship between the BGB/AGB ratio and soil N/P ratio (AGB, aboveground biomass; BGB, belowground biomass)

Figure 5 shows the relationship between biomass and soil N and P levels based on the stepwise linear regression analysis. The mean BGB:AGB ratio declined as the soil N:P ratio increased. When the soil N:P level was >2.0, the fitted curve gradually flattened, implying reduced N:P effects on the biomass (Figure 5).

## 4 Discussion

### 4.1 C:N:P stoichiometry in alpine and arid soils

A previous study found mean soil C:N, C:P, and N:P ratios in the frigid highland zone of 13.6, 62.0, and 5.9, respectively (Tian, Chen, Zhang, Melillo, & Hall, 2010). We found lower C:P (19.87) and N:P (1.97) ratios in studied cryic aridsols of the northern Tibetan Plateau. Frequent freeze–thaw cycles can significantly increase the availability of extractable P in soils at high‐altitude areas (Hinman, 1970), most likely due to the presence of large amounts of aluminum, iron oxide, and highly weathered kaolin clay (McGroddy, Silver, de Oliveira, de Mello, & Keller, 2008). In addition, numerous residues of *Oxytropis DC* found in the study area are high in P and have lower C:P ratios, favoring net P mineralization. A number of studies have confirmed that P net immobilization is most likely to occur when residues added to soils have a C:P ratio >300:1, whereas net mineralization is likely to occur when this ratio is <200:1 (Alamgir, McNeill, Tang, & Marschner, 2012; Brady & Weil, 2002; Huffman, Cole, & Scott, 1996).

Our study results reveal significant and positive associations between total soil C, N, and P levels (Figure 3). Fixed soil C:N ratios across latitudinal gradients found support the fact that plants constitute a major source of total soil C and N in alpine and arid steppe; however, the fixed C:P and N:P ratios found in the soil samples were not anticipated. The latter result indirectly indicates that biological processes, and especially those of P utilization and deposition in topsoil by plants, are more central to the P cycle than contributions of parent materials to the surface soil. Similar results have been found for other alpine regions (Beck & Elsenbeer, 1999; Litaor, Seastedt, Walker, Carbone, & Townsend, 2005). However, some authors have found alpine and arid soils to show low levels of weathering and microbial activity due to the presence of low temperatures in cold alpine climates, where inorganic P constitutes the dominant fraction of soil P and where parent materials control soil P cycling (Cassagne, Remaury, Gauquelin, & Fabre, 2000; Cross & Schlesinger, 1995).

### 4.2 Plant C:N:P ratios in the alpine and arid steppe

Several previous studies found the positive relationship between C and N in plant tissues (Elser, Fagan, Kerkhoff, Swenson, & Enquist, 2010; He et al., 2006). However, our study showed positive correlations between plant C and P, but no clear pattern between C and N (Figure 4). Hidaka and Kitayama (2013) confirmed that the photosynthetic carbon assimilation rate was positively correlated with the concentrations of total foliar P and of metabolic P across 10 tropical species. Much of the evidence in support of P as a key regulator of carbon partitioning has been obtained (Fredeen, Rao, & Terry, 1989; Priya & Sahi, 2009). Our results also implied that P might play an important role in the alpine plant growth.

A number of authors have found average N:P ratios of terrestrial plant species of 12–13 in natural field sites (Elser et al., 2000; Knecht & Goransson, 2004), reflecting the average N:P ratio of aquatic plants and algae (Geider & La Roche, 2002). However, N:P ratios can vary widely, and individual measurements found have roughly ranged from 1 to 100 (Gusewell, 2004; Phoenix et al., 2004; Schmidt, Michelsen, & Jonasson, 1997). The plant N:P ratio has been used to identify limitations between N and P at the community level. Gusewell (2005) proposed that plant N:P ratios <13 and >16 correspond to N and P limitations, respectively, and Tessier and Raynal (2003) also used the leaf N:P ratio as an indicator to determine limitation types, with <14 used for N limitations, with >16 used for P limitations, and with plant growth otherwise being affected by N and P together (Gusewell, 2005; Tessier & Raynal, 2003). Other studies focusing on the eastern Tibetan Plateau have found alpine meadows with leaf N:P ratios of 10.5 to be N limited (Wang et al., 2011). Our results reveal lower N:P ratios in the community (3.50 ± 0.93, Table 2) of the alpine and arid steppe, which seem to imply N limitations of the vegetation. However, the inter‐relationship among P, N, and C indicate that P is more strongly limiting in the alpine plants (Figure 4). It might be that the alpine plants can use P, which is relatively abundant, to protect tissues and membranes against the cold (Marschner, 2012). Plant N:P ratio <14 for N limitation may face challenges in the alpine plants. In the future, more studies should be carried out to understand plant physiological response to N and P limitation in the low‐temperature <5°C regions.

### 4.3 Biomass allocation and soil N:P ratio

Aboveground and belowground biomass levels decreased with increases in latitude (Figure 2a,b), as reduced precipitation and temperature levels heavily affected plant growth. Luo et al. (2013) found similar results for temperate grasslands across a 2,500‐km transect in northern China. However, the below‐/aboveground biomass ratio (nearly 8:1, Table 1) of the alpine and arid steppe, which is positively correlated with latitude (Figure 2c), is larger than that found for global temperate grasslands (4.2; Mokany, Raison, & Prokushkin, 2006). These large belowground biomass stocks can be attributed to nutrient‐poor characteristics of these systems, requiring plants to invest more C in roots to adequately capture available nutrients (Chapin, Bloom, Field, & Waring, 1987). This dominance of belowground plant biomass stock has been found in numerous alpine sites (Hirota et al., 2010; Sebastia, 2007; Zhang et al., 2011). The higher ratio found may be associated with relatively slow carbohydrate depletion from roots, resulting from low respiration rates in these cold regions (Davidson, 1969; Yang et al., 2009) and potentially also from slower root turnover patterns in colder environments (Gill & Jackson, 2000). Several studies have found a decrease in root:shoot ratios with increasing soil temperatures (Delucia, Heckathorn, & Day, 1992; Wilson, 1988). A previous study revealed a decrease in the root:shoot ratio with increasing root temperatures from 5°C to >25°C for 12 pasture species (Davidson, 1969), as higher temperatures increase the root function rate (Thornley, 1972). Furthermore, the effects of rainfall and irradiance on biomass allocation have been studied intensively (Feng & Li, 2007; Sack & Grubb, 2002; Wu et al., 2013).

Biomass allocation patterns in plants are known to be affected by soil nutrient availability. For example, plants show a pattern of decreased allocation to roots as soil nitrogen levels increase (King, 2003; Markham & Zekveld, 2007). Although higher allocation to roots increases access to soil resources, this occurs at the expense of photosynthetic capacity and growth levels (Chapin, 1980). Biomass allocation and soil nutrient supplies are tightly linked to a soil–plant feedback loop. Species with particular mechanisms for the acquisition of either nitrogen or phosphorus tend to dominate in soils where relevant elements are not readily available (Gusewell, 2004). Species with symbiotic N_2_ fixation are generally favored by high phosphorus availability and low nitrogen availability (Vitousek & Field, 1999), and species with effective phosphorus solubilization dominate in soils exhibiting high levels of phosphorus sorption (Lamont, 2003). However, some authors have found that these mechanisms are not necessarily related to biomass allocation (Gusewell, Koerselman, & Verhoeven, 2003; Vance, Uhde‐Stone, & Allan, 2003). In this study, we found strong negative relationships between the soil N:P ratio and BGB:AGB ratio in the Tibetan steppe ecosystem (Figure 5). The fitted curve can be used to predict belowground biomass at this site and in other alpine and arid steppes. This negative correlation indicates that relatively less C should be fixed belowground as the soil N:P ratio increases, especially for an N:P ratio < 2.0. In the future, with increasing soil N:P ratios resulting from nitrogen deposition and excreta returned by grazing animals (Flessa, Dorsch, Beese, Konig, & Bouwman, 1996; Jiang, Yu, Fang, Cao, & Li, 2010), more carbon released into the environment with relatively less carbon fixed under the ground may be expected. The results of our study may supplement quantitative understandings of the synergetic effects of soil nitrogen and phosphorus on alpine plant growth in high‐altitude regions. Furthermore, this study identifies a critical N:P soil ratio of approximately 2.0, above which the probability of BGB:AGB responses to nutrient availability is small.

## 5 Conclusion

A clear and significant decreasing trend in BGB and AGB with latitude levels in the alpine and arid steppe has been determined. However, we observed a nearly linear increase in the BGB:AGB ratio with latitude levels in the northern Tibetan Plateau. Although soil is characterized by structural complexity and spatial heterogeneity, we found remarkably consistent C:N:P ratios in the cryic aridisols examined. In the alpine plants examined, a significant correlation was found among plant phosphorus levels, N:P ratios, and carbon levels at the community level. Therefore, phosphorus still played a key role in maintaining high production levels in naturally N‐limited grassland areas. Our study presents interesting contributions to the global data pool on plant stoichiometry, given previously limited knowledge on high‐altitude regions.

Quantifying root biomass is critical to improving our understanding of carbon cycles and storage in grassland ecosystems. This study offers basic information for use in biomass estimations of the alpine steppe, which covers nearly half of the total Tibetan land area. Furthermore, we found that biomass allocation characterized by the BGB:AGB ratio is heavily affected by soil nutrients. However, more studies are needed to elucidate potential feedback mechanisms between alpine plants and soils.

## Conflict of Interest

None declared.
